# Isolation, identification, and characterization of a marine *Lactiplantibacillus plantarum* strain with antimicrobial activity against *Burkholderia contaminans*

**DOI:** 10.3389/fmicb.2025.1636121

**Published:** 2025-07-22

**Authors:** Yuanshuai Du, Fan Xin, Ziyi Yang, Jiayi Sui, Shen Yang, Runying Zeng, Zhuhua Chan

**Affiliations:** ^1^College of Ocean Food and Biological Engineering, Jimei University, Xiamen, China; ^2^Technology Innovation Center for Exploitation of Marine Biological Resources, Third Institute of Oceanography, Ministry of Natural Resources, Xiamen, China

**Keywords:** marine microorganisms, *Burkholderia contaminans*, *Lactobacillus plantarum*, metabolites, antimicrobial activity

## Abstract

*Burkholderia contaminans*, an opportunistic pathogen commonly found in the food and cosmetics industries, has serious potential to cause severe human infections and industrial contamination. However, compared to traditional physical or chemical antimicrobial treatment, the novel biological antimicrobial strategies against *B. contaminans* have not been extensively explored. In this study, a strain with antimicrobial activity against *B. contaminans* was isolated from a marine grouper aquaculture pond and identified as *Lactobacillus plantarum* Dys01. The antimicrobial activity of *L. plantarum* Dys01 mainly originated from its metabolites, with a minimum inhibitory concentration (MIC) of 8 mg/mL. Component analysis indicated that the antibacterial substances of *L. plantarum* Dys01 primarily included organic acids, proteinaceous substances, and hydrogen peroxide, among which organic acids and proteinaceous substances played the major inhibitory roles. Additionally, the metabolites of *L. plantarum* Dys01 significantly inhibited the biofilm formation of *B. contaminans* in a dose-dependent manner. Alkaline phosphatase activity assays and propidium iodide staining revealed that metabolites produced by *L. plantarum Dys01* could disrupt the cell wall and cell membrane integrity of *B. contaminans.* This was further confirmed by scanning electron microscopy, which showed typical morphological damage such as surface indentations and membrane rupture. Therefore, our study provided novel insights into the control of *B. contaminans* contamination in the food, cosmetic, and pharmaceutical industries, and laid an important theoretical foundation for the development of novel biopreservatives.

## 1 Introduction

*Burkholderia contaminans* is a Gram-negative, aerobic, non-spore-forming, and non-fermentative bacterium that is widely distributed in natural environments such as soil and water (Demirdag et al., 2020). As an opportunistic pathogen, *B. contaminans* poses serious threats to human health, ecological system, and industrial production (Bennett et al., 2020; Ura et al., 2006). In the field of food, *B. contaminans* can lead to food spoilage and foodborne diseases, and is also closely associated with several bacterial food poisoning incidents (Han et al., 2023). In addition, *B. contaminans* is capable of producing various toxins that can cause infectious diseases, such as septicemia and pneumonia, with an increased risk for immunocompromised individuals (Nunvar et al., 2016). In the cosmetics industry, *B. contaminans* can proliferate extensively by utilizing the nutrients in cosmetic formulations, thereby compromising product stability and leading to visible changes in the texture, color, and odor of cosmetics. Exposure to contaminated cosmetics may trigger adverse skin reactions, including erythema, pruritus, and rashes (Akhand et al., 2023). Due to these potential risks, regulatory agencies such as those in the United States and the European Union have classified *B. contaminans* as an unacceptable microorganism in cosmetic products, mandating that this bacterium must not be detected in finished products (Tavares et al., 2020).

In industrial production processes, improper hygiene management or equipment contamination can allow *B. contaminans* to enter the product system through water sources, production lines, or packaging materials, leading to significant safety risks. *B. contaminans* exhibits strong resistance to conventional disinfection methods, making it difficult to eliminate thoroughly (Li et al., 2023). For example, *B. contaminans* has been shown to resist the majority of antibiotics including *β*-lactams, third- and fourth-generation cephalosporins and fluoroquinolones (Lama et al., 2021). Moreover, some *Burkholderia* species display resistance to disinfectants commonly used in clinical settings such as Hibiscrub, with some strains remaining viable after 1 h of exposure of Hibiscrub (Rose et al., 2009). Currently, control strategies for *B. contaminans* mainly include: (1) physical methods, such as heat or irradiation; (2) chemical or pharmaceutical approaches, including the use of antimicrobials such as minocycline and the combination of trimethoprim-sulfamethoxazole. However, these approaches have notable limitations (McGowan, 2006). Excessive heat or irradiation may affect product quality, while chemical/pharmaceutical treatment may result in incomplete sterilization. The use of antibiotics is generally restricted to medical applications and is unsuitable for food and cosmetics. Most importantly, the misuse of antibiotics has exacerbated the development of antimicrobial resistance (Reddy and Love, 1999; Bacanlı, 2024). Studies have shown that the genetic structure of resistant bacteria can evolve rapidly, rendering conventional antibiotic therapies ineffective within a few years (Muteeb et al., 2023). Therefore, in response to the threats caused by *B. contaminans*, it is imperative to develop efficient, safe, and cost-effective antimicrobial strategies to meet the diverse application demands of various fields. In recent years, biological antimicrobial approaches, particularly those involving antagonistic microorganisms, have attracted widespread attention due to their ecological safety and low resistance risk. However, the bacteria which can effectively inhibit *B. contaminans* remain largely unclear.

*Lactiplantibacillus plantarum* is a widely studied probiotic bacterium with multiple functional properties, including antimicrobial activity, gut microbiota modulation, immunoregulation, and antioxidant effects (Aziz et al., 2023). It is extensively applied in the food, medicine, probiotics, and agriculture. Compared with traditional physical, chemical, and pharmaceutical antimicrobial strategies, *L. plantarum* offers superior biocompatibility and safety (Pradhan et al., 2019). Recently, *L. plantarum and* its metabolites have received more attentions due to their antimicrobial activity, making them become potential candidates for novel biological antimicrobial agents (Rocchetti et al., 2021). In particular, marine-derived *L. plantarum* and other lactic acid bacteria (LABs) are expected to possess superior antimicrobial potential compared to terrestrial strains, as they have adapted to harsh marine conditions such as high salinity, pressure, and low temperatures (Stincone and Brandelli, 2020). These environmental pressures drive the synthesis of unique metabolites with enhanced stability and bioactivity (De Carvalho and Fernandes, 2010), as exemplified by an isolated marine strain *Lactobacillus plantarum* EI6, which exhibits broad-spectrum antimicrobial activity and tolerance to low pH and high salt concentrations (Zaghloul and Ibrahim, 2022). The antimicrobial activity of *L. plantarum* is mainly attributed to its metabolites, including organic acids (Rao et al., 2024), fatty acids (Kong et al., 2021), hydrogen peroxide (Wang et al., 2022), and bacteriocins (Yilmaz et al., 2022). These metabolites exhibit significant inhibitory effects against various microorganisms. For example, bacteriocins are ribosomally synthesized peptides or proteins with broad-spectrum antibacterial activity, and serve as the key antimicrobials in LABs (Darbandi et al., 2022; Rocchetti et al., 2021). The bacteriocins from *L. plantarum* are not only harmless to the human body (Leuschner et al., 2010), but also effectively inhibit the growth of pathogens, such as *Listeria monocytogenes*, *Escherichia coli*, and *Staphylococcus aureus*, making them promising natural biological preservatives for ensuring food safety and extending shelf life without relying on synthetic chemicals (Wang et al., 2018; Hussein et al., 2025). The lactic acid and other organic acids metabolized by *L. plantarum* can suppress common foodborne pathogenic bacteria such as *Salmonella enterica* and *Listeria monocytogenes* (Arena et al., 2016). Furthermore, the metabolites of *L. plantarum* exhibit antifungal activity against mycotoxigenic fungi such as *Aspergillus flavus* and *Aspergillus ochraceus* (Shehata et al., 2019), as well as spoilage bacteria in aquatic products, including *Vibrio alginolyticus, Aeromonas hydrophila* (Sharma et al., 2022). These metabolites may function independently or synergistically through multiple mechanisms to inhibit the growth of pathogens, thereby enhancing the safety and shelf life of food and cosmetic products (Tsai et al., 2021). However, the antimicrobial activity of *L. plantarum* against *B. contaminans* remains unclear.

In this study, a strain with antimicrobial activity against *B. contaminans* was isolated from a marine grouper aquaculture pond and identified as *L. plantarum* Dys01. The antimicrobial activity of this strain and its metabolites was evaluated, followed by analysis of the main antimicrobial components and antimicrobial mechanisms. This study offers novel insights into the development of *L. plantarum* as a natural and effective biopreservative for the control of *B. contaminans* contamination.

## 2 Materials and methods

### 2.1 Isolation and identification of strains against *Burkholderia contaminans*

Fresh intestinal tissue from grouper and sediment samples from marine grouper aquaculture ponds were collected. The intestinal tissues were homogenized using a tissue grinder, and stored at 4°C for further strain isolation. 1 mL of each sample was serially diluted (10^6^ to 10^8^) using sterile saline. A 100 μL aliquot from each dilution was spread onto MRS agar (HuanKai Microbial Technology Co., Ltd., China) plates and incubated at 37°C for 48 h. The single colonies were selected, purified by repeated streaking, and then inoculated into MRS liquid medium for 48 h. The antimicrobial activity of the isolates was tested using the Oxford cup assay. Briefly, the indicator strain *B. contaminans* was mixed with nutrient agar (HuanKai Microbial Technology Co., Ltd., China) at a 1:25 ratio at 45°C, poured into plates containing Oxford cups, and after solidification, 200 μL of each bacterial culture was added to the cups. After 48 h of incubation at 37°C, the diameters of the inhibition zones were measured to assess the inhibitory effects of the strains against *B. contaminans.*

To identify the strain with significant antibacterial activity, bacterial DNA was extracted using the Bacterial genomic DNA extraction kit (Vazyme, China) following manufacturer’s protocols. The extracted DNA was subjected to bacterial 16S rRNA sequencing using universal bacterial primers (27F, 5′-AGAGTTTGATCCTG GCTCAG-3′; 1492R, 5′-GGTTACCTTGTTACGACTT-3′). The PCR reaction mixture (50 μL) contained 25 μL 2 × PCR buffer (Vazyme, China), 1 μL dNTPs (10 μM) (Vazyme, China), 2 μL of each primer (10 μM), 1 μL DNA polymerase (Vazyme, China), and 1 μL template DNA. PCR conditions were as follows: initial denaturation at 98°C for 10 min, followed by 30 cycles of denaturation at 95°C for 20 s, annealing at 50°C for 20 s, and extension at 72°C for 30 s, with a final extension at 72°C for 5 min. The PCR products were sequenced by Xiamen Borui Biotechnology Co., Ltd. The 16S rRNA gene sequences were analyzed using BLAST to compare homology with known bacterial strains. Phylogenetic analysis was conducted using MEGA11 software to construct a neighbor-joining tree.

### 2.2 Preparation of *Lactiplantibacillus plantarum* Dys01 metabolites and *Burkholderia contaminans* suspension

*L. plantarum* Dys01 was activated in MRS liquid medium for optimal viability and then inoculated into fresh MRS medium at a 2% (v/v) ratio. The bacterium was cultured at 37°C for 16–18 h, followed by centrifugation at 8,000 rpm for 10 min at 4°C. The supernatant, containing *L. plantarum* Dys01 metabolites, was collected, and filtered through a 0.22 μm sterile membrane to remove remaining bacteria. Then the supernatant was either used directly or subjected to lyophilization for later use and determination of metabolite concentrations. *B. contaminans*, used as the indicator strain, was activated on nutrient agar. A single colony was inoculated into 5 mL of sterile nutrient broth (10.0 g/L peptone, 3.0 g/L beef extract powder, 5.0 g/L sodium chloride, final pH 7.3 ± 0.2) and incubated at 37°C for 18–24 h. The culture was then diluted with sterile nutrient broth to a final concentration of 1–2 × 10^7^ CFU/mL for subsequent assays.

### 2.3 Determination of the minimum inhibitory concentration (MIC) of *Lactiplantibacillus plantarum* Dys01 metabolites

The minimum inhibitory concentration (MIC) of *L. plantarum Dys01* metabolites against *B. contaminans* was determined using a two-fold serial dilution method modified from Zhou et al. (2019). Briefly, in a 96-well microtiter plate, each well was loaded with 100 μL of *B. contaminans* suspension prepared in nutrient broth and 100 μL of *L. plantarum* Dys01 metabolites prepared in sterile deionized water at final concentrations of 32, 16, 8, 4, 2, 1, 0.5, and 0.25 mg/mL, with the metabolite concentrations determined by the lyophilized *L. plantarum* Dys01 metabolites. As a control, 100 μL sterile deionized water was added in *B. contaminans* suspension. Plates were incubated at 37°C for 24 h. Bacterial growth was assessed visually by observing turbidity. The minimum concentration of *L. plantarum* Dys01 metabolites that completely cleared the *B. contaminans* suspension was defined as the minimum inhibitory concentration (MIC) of the *L. plantarum* Dys01 metabolites against the *B. contaminans*.

### 2.4 Analysis of the antimicrobial components in the metabolites of *Lactiplantibacillus plantarum* Dys01

To identify the key antimicrobial components in the cell-free supernatant (CFS) of *L. plantarum* Dys01 responsible for inhibiting *B. contaminans*, three different treatments were applied respectively: (1) catalase (10 U/mL) was added to degrade hydrogen peroxide; (2) acidic protease (200 μg/mL) was used to break down proteinaceous antimicrobial substances; and (3) the CFS was adjusted to pH 7.0 using 1 M NaOH to neutralize the effect of organic acids. After treatment, each sample was co-incubated with *B. contaminans* in nutrient broth at 37°C for 12 h. Antibacterial activity was evaluated by measuring the OD₆₀₀ of the culture.

### 2.5 Effect of *Lactiplantibacillus plantarum* Dys01 metabolites on biofilm formation of *Burkholderia contaminans*

The influence of *L. plantarum* Dys01 metabolites on *B. contaminans* biofilm formation was assessed using a 96-well microtiter plate assay. Each well was filled with 100 μL of *B. contaminans* suspension prepared in nutrient broth and 100 μL of *L. plantarum* Dys01 motabolite solution prepared in sterile deionized water at final metabolite concentrations of 0.5 × MIC, 1.0 × MIC, and 2.0 × MIC. The control group was supplemented with 100 μL of *B. contaminans* and 100 μL of sterile deionized water. After 24 h incubation at 37°C to allow biofilm development, the wells were gently washed three times with phosphate-buffered saline (PBS) to remove planktonic bacteria. Biofilms were fixed with 200 μL of methanol for 15 min, followed by methanol removal. Then, 200 μL of 0.1% crystal violet was added for 20 min of staining. Excess dye was removed by PBS washing, and plates were air-dried. Finally, 200 μL of 95% ethanol was added to dissolve the retained dye. The absorbance at 590 nm for each well was measured using a microplate reader, and the biofilm inhibition effect was evaluated by calculating the absorbance values. The biofilm inhibition rate was calculated as follows:


$$\text{Biofilm inhibition rate}\;(\%)=\frac{{\mathrm{OD}}_{\text{control}}-{\mathrm{OD}}_{\text{treatment}}}{{\mathrm{OD}}_{\text{control}}}\times 100\%$$

### 2.6 Effect of *Lactiplantibacillus plantarum* Dys01 metabolites on the cell wall integrity of *Burkholderia contaminans*

1 mL of *B. contaminans* suspension in nutrient broth was mixed with 1 mL of *L. plantarum* Dys01 metabolites prepared in water, achieving final metabolite concentrations of 0.5 ×, 1 ×, and 2 × MIC. Sterile deionized water was used as a control. After incubation at 37°C for 4 h, the mixtures were centrifuged at 8,000 rpm for 15 min at 4°C, and then the resulting supernatants were collected for the detection of alkaline phosphatase (AKP) activity by AKP assay kit (Nanjing Saihongrui Biotechnology Co., Ltd., China) to assess the cell wall integrity of *B. contaminans*.

### 2.7 Effect of *Lactiplantibacillus plantarum* Dys01 metabolites on the cell membrane integrity of *Burkholderia contaminans*

1 mL of *B. contaminans* suspension was mixed with *L. plantarum* Dys01 metabolites to reach final metabolite concentrations of 0.5 ×, 1 ×, and 2 × MIC. Sterile deionized water was used as a control. After incubation at 37°C for 4 h, the samples were centrifuged at 8,000 rpm for 15 min at 4°C, and the bacterial pellets were collected. The pellets were washed three times with PBS and then resuspended in PBS. For cell membrane integrity assessment, the bacteria were stained with propidium iodide (PI, 10 μg/mL) in the dark for 30 min. After staining, cells were washed twice with PBS and resuspended in PBS. A 10 μL aliquot of each sample was observed under a fluorescence microscope.

### 2.8 Field emission scanning electron microscopy observation

10 mL of *B. contaminans* suspension was mixed with 10 mL of *L. plantarum* Dys01 metabolites at final concentrations of 0.5 ×, 1 ×, and 2 × MIC in sterile 50 mL centrifuge tubes. Sterile deionized water was used as a control. After incubation at 37°C for 12 h, samples were centrifuged at 8,000 rpm for 15 min, and the bacterial pellets were collected. After washing by PBS, the pellets were fixed overnight at 4°C with 2.5% glutaraldehyde. Samples were post-fixed with 1% osmium tetroxide for 30 min, dehydrated through a graded ethanol series, dried using a critical point dryer, and gold-coated using a sputter coater. The morphological changes of *B. contaminans* were observed using a field emission scanning electron microscopy (FESEM).

### 2.9 Statistical analysis

All experiments were performed with at least three biological replicates, each including at least three technical replicates. All numerical data were presented as the mean ± standard deviation. Statistical significance analysis of numerical data was performed using Student’s t-test, and the analysis was carried out using Origin 2021 software. The *p* values (P) < 0.05 are considered to indicate a statistically significant difference.

## 3 Results

### 3.1 Isolation and identification of *Lactiplantibacillus plantarum* Dys01 with antimicrobial activity against *Burkholderia contaminans*

**Figure 1:**
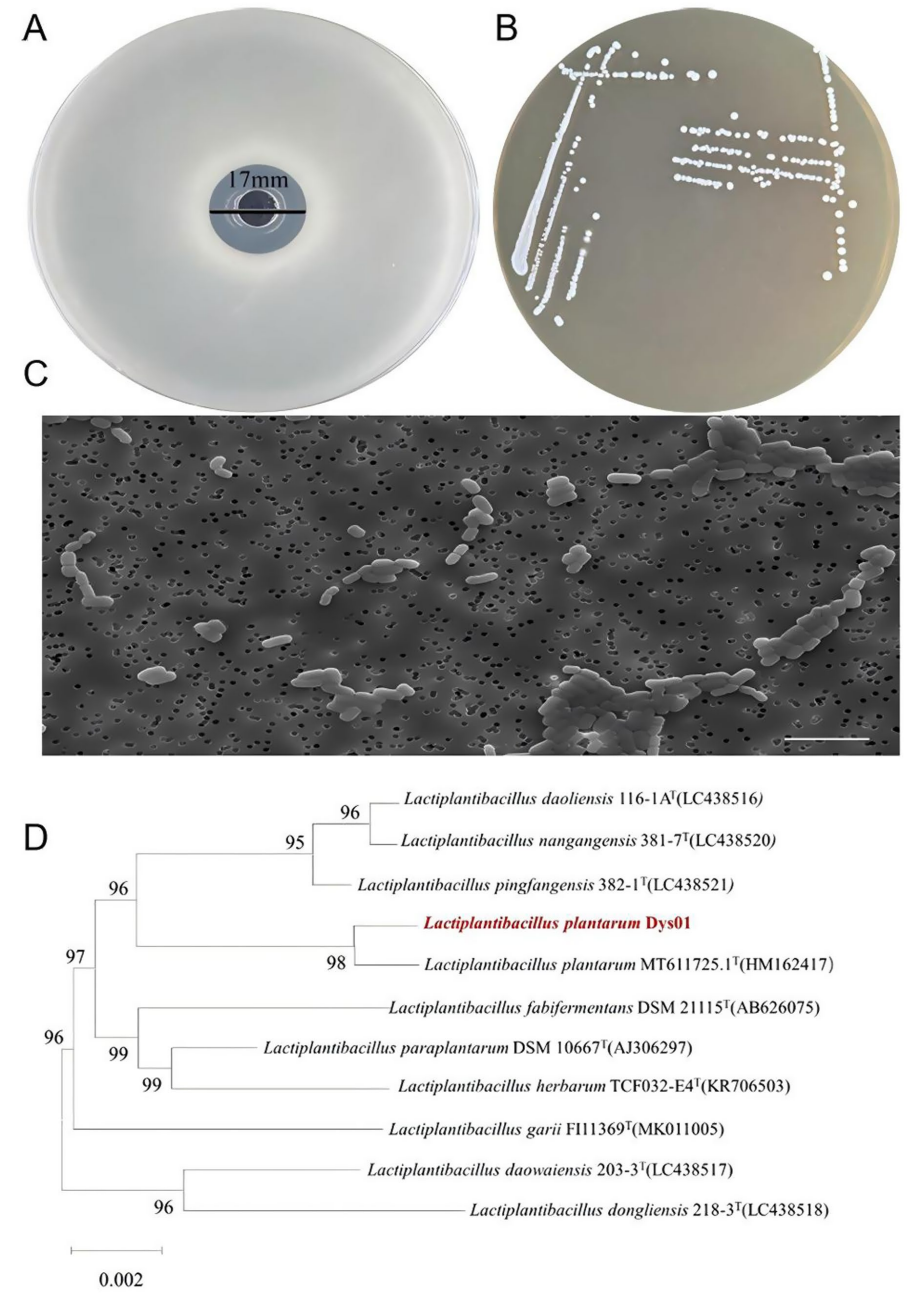
Isolation and identification of *L. plantarum* Dys01 with antimicrobial activity against *B. contaminans*. **(A)** Inhibition zone analysis of *L. plantarum* Dys01 against *B. contaminans* determined by the Oxford cup assay. **(B)** Colony morphology of *L. plantarum* Dys01 on MRS agar. **(C)** Transmission electron microscopy analysis of *L. plantarum* Dys01. Scale bar, 10 μm. **(D)** Neighbor-joining phylogenetic tree of *L. plantarum* Dys01 and the related bacteria according to 16S rRNA gene sequences. The position of *L. plantarum* Dys01 in the tree is indicated with red color. The tree was generated by MEGA11 with a bootstrap value of 1,000.

To identify bacteria with antimicrobial activity against *B. contaminans*, bacterial strains were isolated from the intestines of marine grouper and sediment from a grouper aquaculture pond using MRS medium. A total of 17 strains were obtained, with 11 strains isolated from grouper intestines, labeled as Dys01–Dys11, and 6 strains from the sediment of marine aquaculture pond, labeled as N0–N6 (Table 1). These strains were screened for antibacterial activity against *B. contaminans* using the Oxford cup assay. Among them, strain Dys01 showed the most significant inhibitory effect against *B. contaminans*, with an inhibition zone diameter of 17 ± 0.23 mm (Table 1; Figure 1A). Therefore, strain Dys01 was chosen for further study.

**Table 1:** Isolation and identification strains with antimicrobial activity against *B. contaminans.*

Strain ID	Source	Inhibition zone diameter (mm)
Dys01	Intestine	17 ± 0.11
Dys02	Intestine	11 ± 0.21
Dys03	Intestine	10 ± 0.11
Dys04	Intestine	11 ± 0.15
Dys05	Intestine	8 ± 0.16
Dys06	Intestine	10 ± 0.30
Dys07	Intestine	12 ± 0.26
Dys08	Intestine	0
Dys09	Intestine	0
Dys10	Intestine	3 ± 0.24
Dys11	Intestine	5 ± 0.21
N01	Sediment	0
N02	Sediment	0
N03	Sediment	3.5 ± 0.32
N04	Sediment	4.2 ± 0.51
N05	Sediment	6.3 ± 0.42
N06	Sediment	0

To identify strain Dys01, this strain was cultured on MRS medium, followed by observation of its morphological characteristics. The results showed that the colonies of strain Dys01 were round and small in shape, white or off-white in color, with a smooth and moist surface, and exhibited some stickiness (Figure 1B). Transmission electron microscopy analysis revealed that strain Dys01 exhibited a rod-shaped morphology with a mean length of 3 μm and a mean width of 1 μm (Figure 1C). The above characteristics were similar to those of the genus *Lactiplantibacillus* (Corsetti and Gobbetti, 2003). To further establish the taxonomic classification of strain Dys01, the 16S rRNA gene of strain Dys01 was sequenced and subjected to phylogenetic analysis. The results indicated that strain Dys01 belonged to the genus *Lactiplantibacillus* and clustered with *Lactiplantibacillus plantarum* MT611725.1^T^ (Figure 1D). A comparison in the GenBank database showed a 99.93% similarity in the 16S rRNA sequence between strain Dys01 and *L. plantarum* MT611725.1^T^. Taken together, based on the morphological and phylogenetic analyses, strain Dys01 was identified as *L. plantarum* Dys01, with antimicrobial activity against *B. contaminans*.

### 3.2 Antimicrobial effect and minimum inhibitory concentration of *Lactiplantibacillus plantarum* Dys01 metabolites against *Burkholderia contaminans*

**Figure 2:**
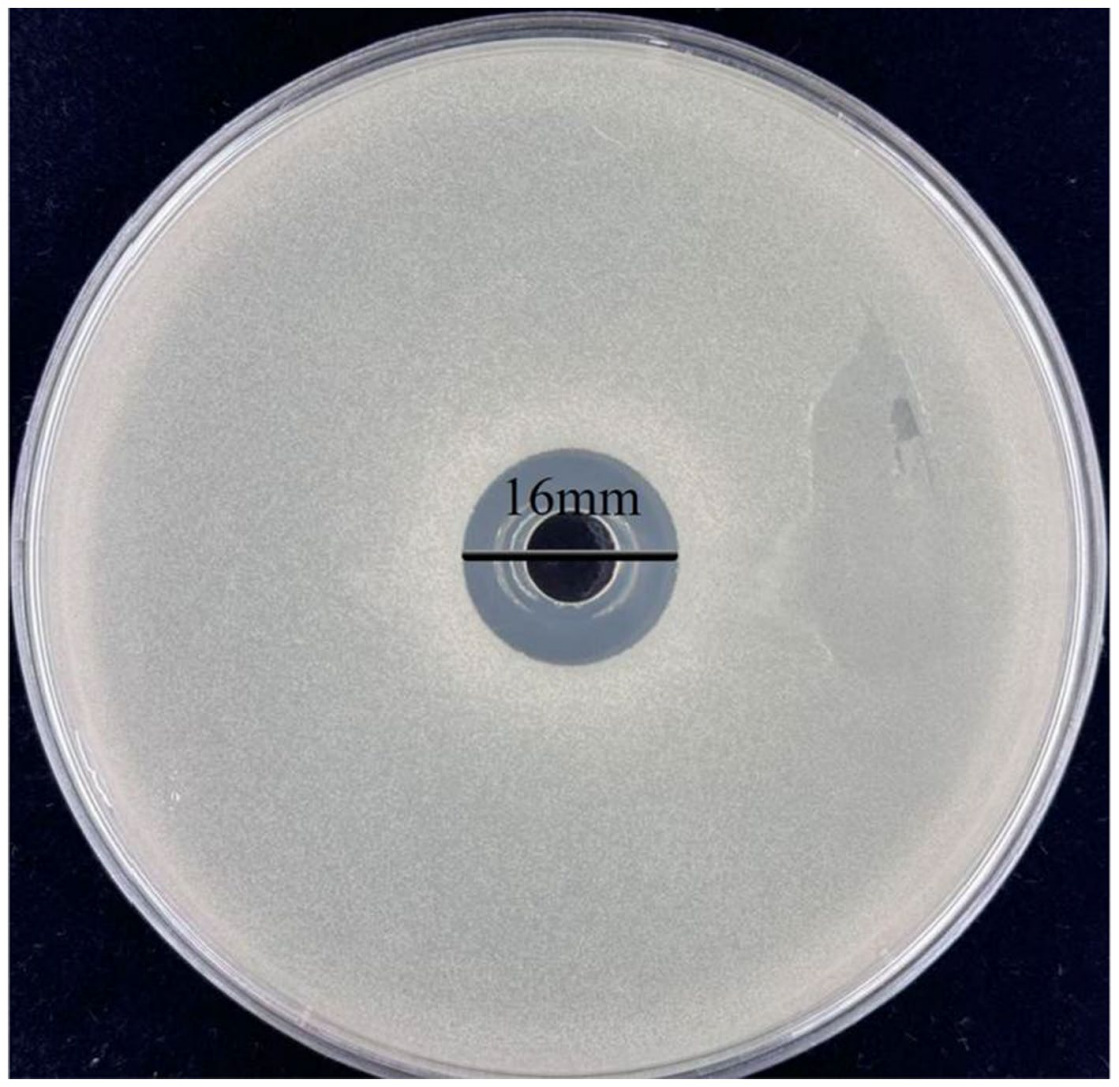
Antimicrobial activity of *L. plantarum* Dys01 metabolites against *B. contaminans*. The metabolites of *L. plantarum* Dys01 were extracted and added to plates containing *B. contaminans*, the antimicrobial activity of metabolites was evaluated by inhibition zone.

Previous studies have reported that the antimicrobial activity of lactic acid bacteria is largely attributed to their metabolic products, such as organic acids, hydrogen peroxide, and bacteriocins (Wang et al., 2022). To evaluate the antibacterial effect of *L. plantarum* Dys01 metabolites on *B. contaminans*, the metabolites of *L. plantarum* Dys01 were extracted and added to plates containing *B. contaminans* for antibacterial activity assay, the results showed the metabolites of *L. plantarum* Dys01 exhibited a significant inhibitory effect on *B. contaminans*, with an inhibition zone diameter of 16 ± 0.24 mm, which was similar to the inhibition effect produced by *L. plantarum* Dys01 itself (Figure 2), indicating that the antibacterial effect of *L. plantarum* Dys01 was mainly attributable to its metabolites. The minimum inhibitory concentration (MIC) assays of *L. plantarum* Dys01 metabolites against *B. contaminans* showed that at a metabolites concentration of 4 mg/mL, *B. contaminans* still grew significantly in the liquid medium, resulting in turbidity in medium (Table 2). However, when the concentration of *L. plantarum* Dys01 metabolites was increased to 8 mg/mL, the medium stayed clear during cultivation, indicating that the growth of *B. contaminans* was significantly inhibited (Table 2). Therefore, the MIC of *L. plantarum* Dys01 metabolites against *B. contaminans* was 8 mg/mL.

**Table 2:** Minimum inhibitory concentration (MIC) of *L. plantarum* Dys01 metabolites against *B. contaminans*.

Concentration of *L. plantarum* Dys01 metabolites (mg/mL)	0	0.25	0.5	1	2	4	8	16	32
Growth status of *B. contaminans* (+/−)	+	+	+	+	+	+	−	−	−

“+” indicates turbidity; “−” indicates clarity.

### 3.3 Antimicrobial component analysis of *Lactiplantibacillus plantarum* Dys01 metabolites

**Figure 3:**
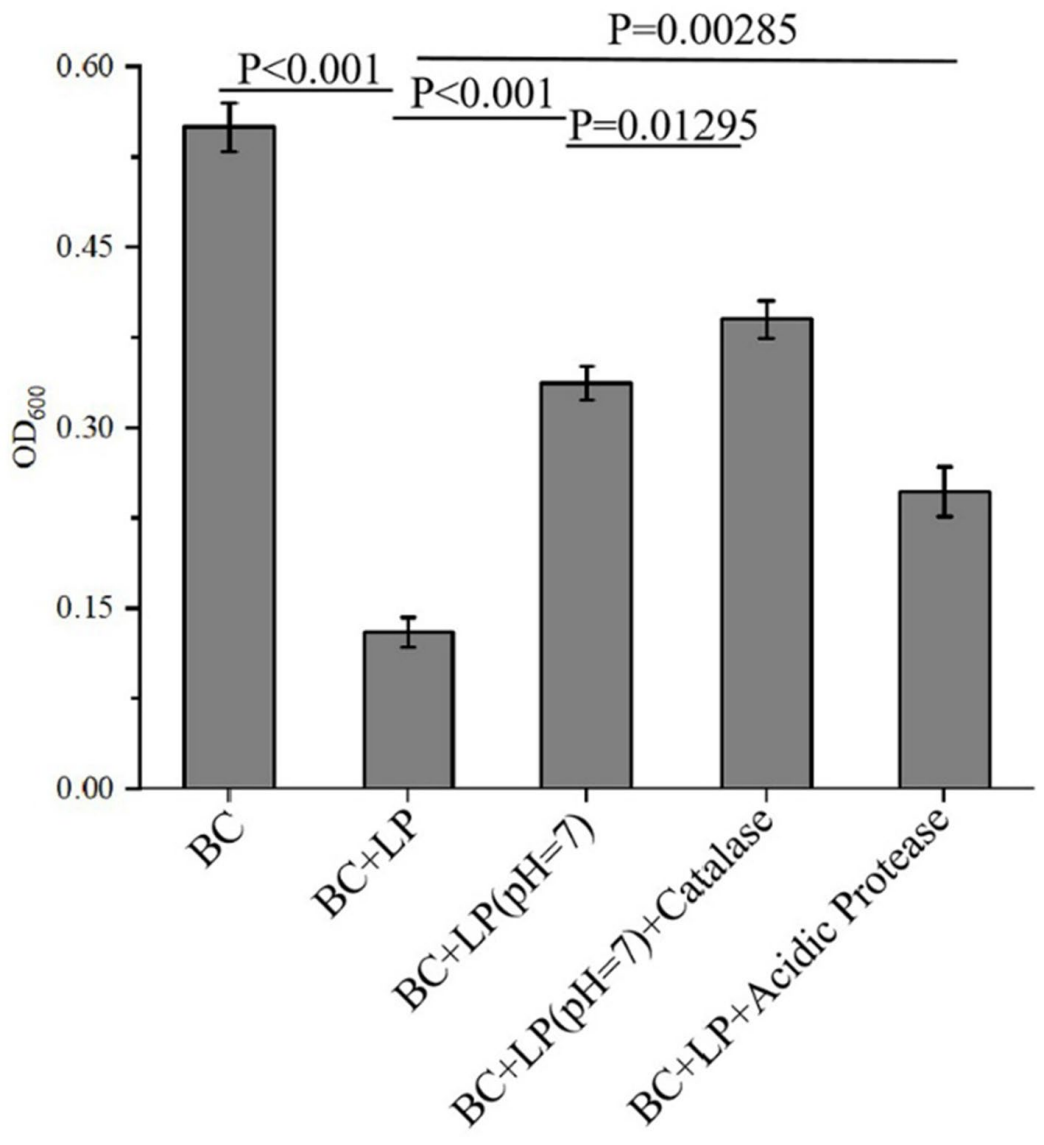
Antimicrobial component analysis of *L. plantarum* Dys01 metabolites against *B. contaminans*. The *L. plantarum* Dys01 metabolites were treated with NaOH, catalase or acidic protease, respectively. After treatment, each sample was co-incubated with *B. contaminans* for 12 h, followed by detection of OD_600_. BC, *B. contaminans* suspension without the addition of any metabolites; BC + LP, the control group treated with *L. plantarum* Dys01 metabolites without any treatment; BC + LP (pH 7.0), the group treated with pH-neutralized metabolites (adjusted to pH 7.0); BC + LP (pH 7.0) + Catalase, the group treated with catalase-treated metabolites (to eliminate hydrogen peroxide); BC + LP + Acidic Protease, the group treated with acidic protease-treated metabolites (to digest proteinaceous substances).

As reported, the antimicrobial activity of the bacterial metabolites may result from the various antimicrobial components they contain, such as organic acids, hydrogen peroxide, proteinaceous substances, or other metabolic byproducts (Fuochi et al., 2019). To identify the active components in the metabolites of *L. plantarum* Dys01 responsible for the inhibition of *B. contaminans*, the effects of organic acids, proteinaceous antimicrobial substances, and hydrogen peroxide from *L. plantarum* Dys01 metabolites were investigated. The results showed that after 12 h of culture with the metabolites of *L. plantarum* Dys01, the growth of *B. contaminans* was significantly inhibited, with the OD_600_ value decreasing to 0.13 (Figure 3). When organic acids were neutralized by adjusting the metabolites of *L. plantarum* Dys01 to pH 7.0 using NaOH, the antibacterial effect was significantly reduced, with the OD₆₀₀ increasing to 0.337 and the inhibition rate decreasing by 37.63%, suggesting that organic acids in *L. plantarum* Dys01 metabolites played a key role in the inhibition of *B.contaminans* (Figure 3). To estimate the effect of proteinaceous substances on the growth of *B. contaminans*, the *L. plantarum* Dys01 metabolites were treated with acidic protease to digest the proteins, and then co-cultured with *B. contaminans*. The results indicated the digestion of proteinaceous substances in *L. plantarum* Dys01 metabolites significantly attenuated its antimicrobial effect on *B. contaminans*, with the inhibition rate decreasing from 76.36 to 55.09%, suggesting that proteinaceous substances are among the key antimicrobials (Figure 3). For the effect of hydrogen peroxide, given that the optimal activity of catalase occurred at pH 7.0 (Trawczyńska, 2020), the metabolites were first adjusted to pH 7.0, and then treated with catalase to remove hydrogen peroxide. Compared to the metabolites at pH 7.0 without catalase treatment, catalase-treated metabolites showed a slight reduction in inhibitory activity on *B. contaminans*, with an OD₆₀₀ of 0.39 and a 9.6% decrease in inhibition rate (Figure 3).

Taken together, these findings demonstrated that the organic acids, proteinaceous substances, and hydrogen peroxide from *L. plantarum* Dys01 metabolites had important roles in inhibiting *B. contaminans*, with organic acids and proteinaceous substances being the primary contributors.

### 3.4 Effect of *Lactiplantibacillus plantarum* Dys01 metabolites on biofilm formation of *Burkholderia contaminans*

**Figure 4:**
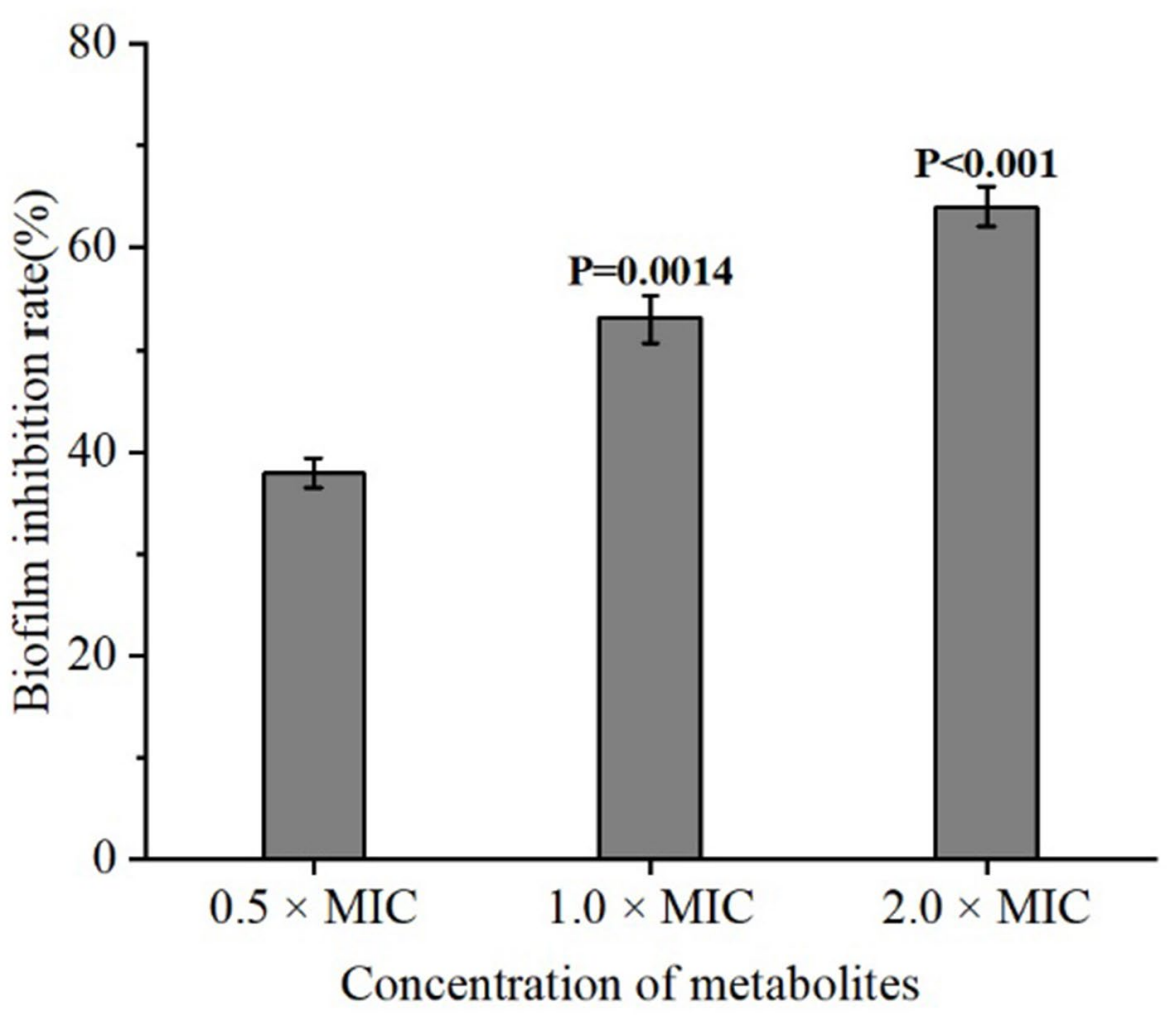
Inhibitory effect of *L. plantarum* Dys01 metabolites on *B. contaminans* biofilm formation. The *L. plantarum* Dys01 metabolites were mixed with *B. contaminans* suspension at final metabolite concentrations corresponding to 0.5 ×, 1 ×, and 2 × MIC. After incubation for 4 h, the biofilm inhibition rate was ascertained by crystal violet assays.

Bacterial biofilms are complex structures embedded in an extracellular matrix and play important roles in the resistance to bacteriophages, chemical disinfectants and antibiotics (Cheng et al., 2022). To assess whether the metabolites of *L. plantarum* Dys01 could affect biofilm formation of *B. contaminans*, the biofilm inhibition assays were performed. The results showed that compared to the control group without any treatment, the addition of *L. plantarum* Dys01 metabolites at a concentration of 0.5 × MIC significantly inhibited *B. contaminans* biofilm formation with the inhibition rate of 37.3% (Figure 4). As the concentration increased, the inhibitory effect on *B. contaminans* biofilm formation was enhanced, with the biofilm inhibition rate reaching 52.2% at 1.0 × MIC and 65.2% at 2.0 × MIC. These findings indicated that the metabolites of *L. plantarum* Dys01 could inhibit *B. contaminans* biofilm formation in a dose-dependent manner.

### 3.5 Effect of *Lactiplantibacillus plantarum* Dys01 metabolites on the cell wall integrity of *Burkholderia contaminans*

**Figure 5:**
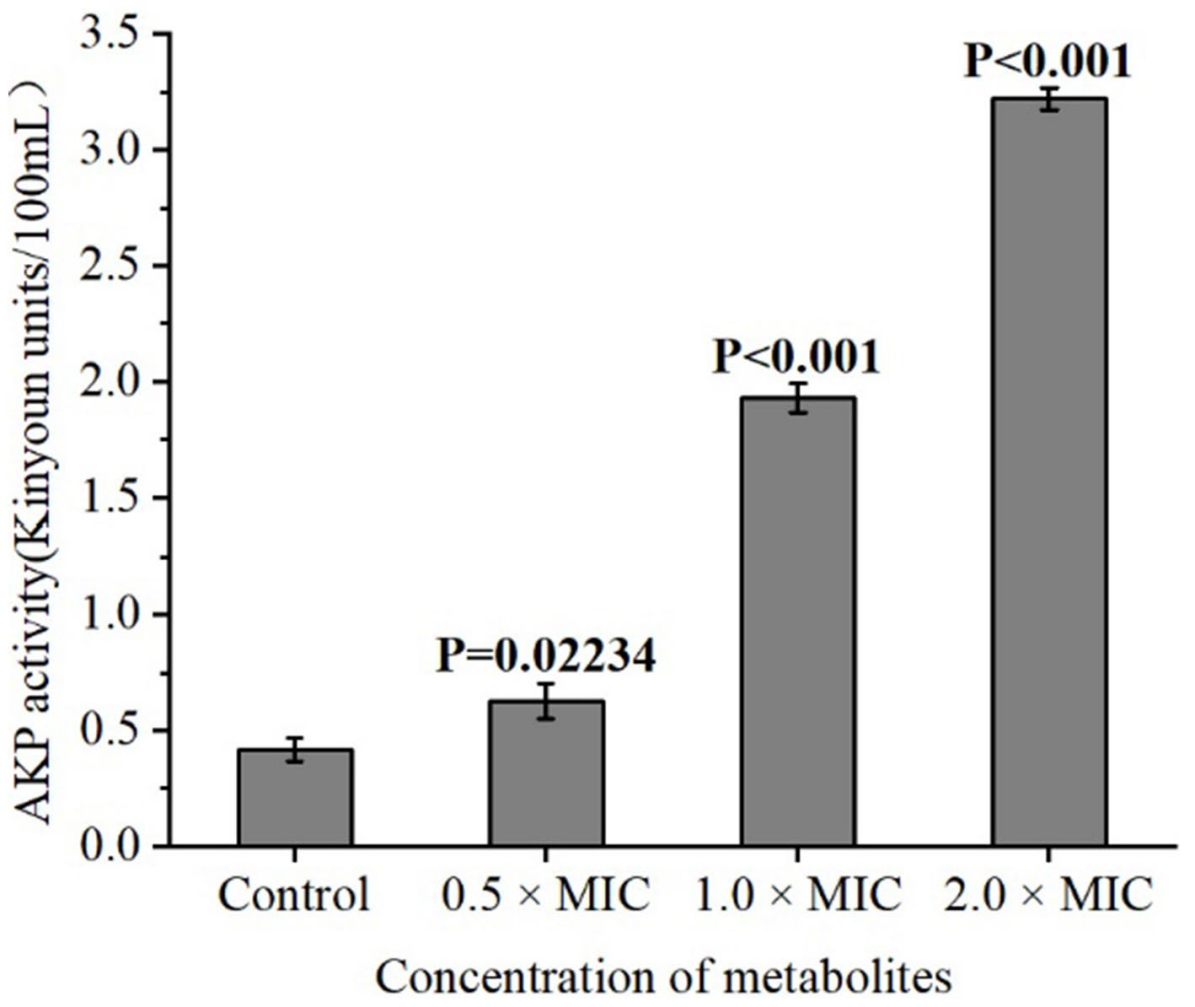
Effect of *L. plantarum* Dys01 metabolites on the cell wall integrity of *B. contaminans.* The *L. plantarum* Dys01 metabolites were mixed with *B. contaminans* suspension at final metabolite concentrations of 0.5 ×, 1 ×, and 2 × MIC. The cell wall integrity of *B. contaminans* was assessed by alkaline phosphatase (AKP) activity assays. The sterile deionized water was employed as a control.

To investigate the effect of different concentrations of *L. plantarum* Dys01 metabolites on the cell wall integrity of *B. contaminans*, the activity of alkaline phosphatase (AKP), a periplasmic enzyme served as an indicator of cell wall damage, was measured in the supernatant of *B. contaminans* culture medium. The results showed that when treated with 1.0 × MIC or 2.0 × MIC of *L. plantarum* Dys01 metabolites, the AKP activity in the supernatant of *B. contaminans* culture medium was significantly enhanced compared to the control group, while the metabolites at low concentrations (0.5 × MIC) slightly upregulated the AKP activity (Figure 5). These findings suggested that *L. plantarum* Dys01 metabolites could impair the cell wall integrity of *B. contaminans* in a dose-dependent manner, resulting in the increased AKP activity in the supernatant of *B. contaminans* culture medium.

### 3.6 Effect of *Lactiplantibacillus plantarum* Dys01 metabolites on the cell membrane integrity of *Burkholderia contaminans*

**Figure 6:**
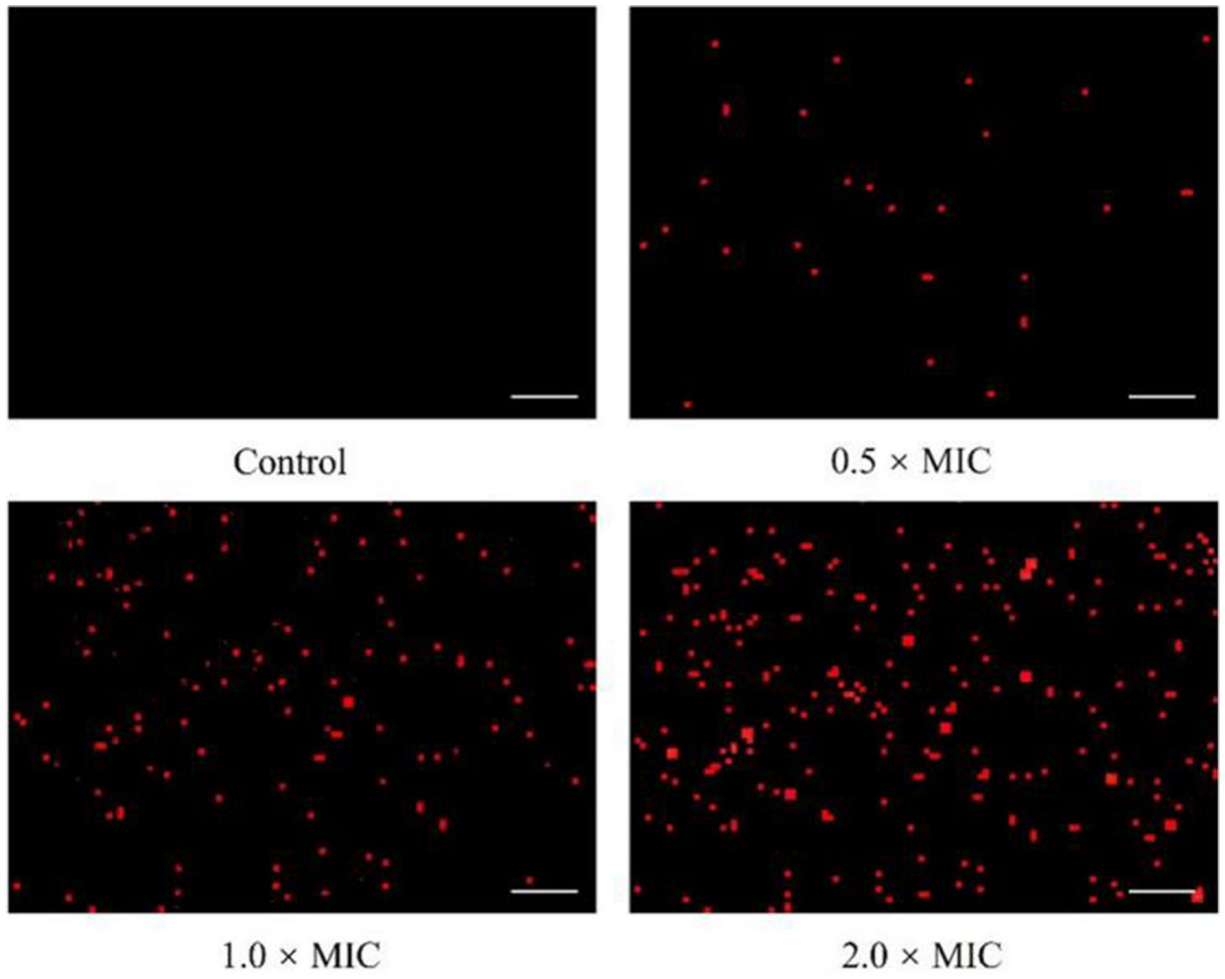
Effect of *L. plantarum* Dys01 metabolites on the cell membrane integrity of *B. contaminans*. The *B. contaminans* treated with *L. plantarum* Dys01 metabolites at final metabolite concentrations of 0.5 ×, 1 ×, and 2 × MIC were labeled with propidium iodide (red) and then subjected to confocal microscopy analysis. The bacteria with damaged cell membranes were stained red. The sterile deionized water was employed as a control. Scale bar, 20 μm.

To explore the influence of *L. plantarum* Dys01 metabolites on the cell membrane integrity of *B. contaminans*, propidium iodide (PI), a fluorescent dye widely used to stain bacteria with damaged cell membranes red, was employed to assess the cell membrane integrity of *B. contaminans*. In the untreated control, almost no fluorescence signal was detected, while the treatment of 0.5 × MIC *L. plantarum* Dys01 metabolites led to faint red fluorescence in *B. contaminans* (Figure 6). Moreover, the fluorescence intensity increased with higher metabolite concentrations (Figure 6). Consequently, the metabolites of *L. plantarum* Dys01 could impair the cell membrane integrity of *B. contaminans*.

### 3.7 Effect of *Lactiplantibacillus plantarum* Dys01 metabolites on the morphology of *Burkholderia contaminans*

**Figure 7:**
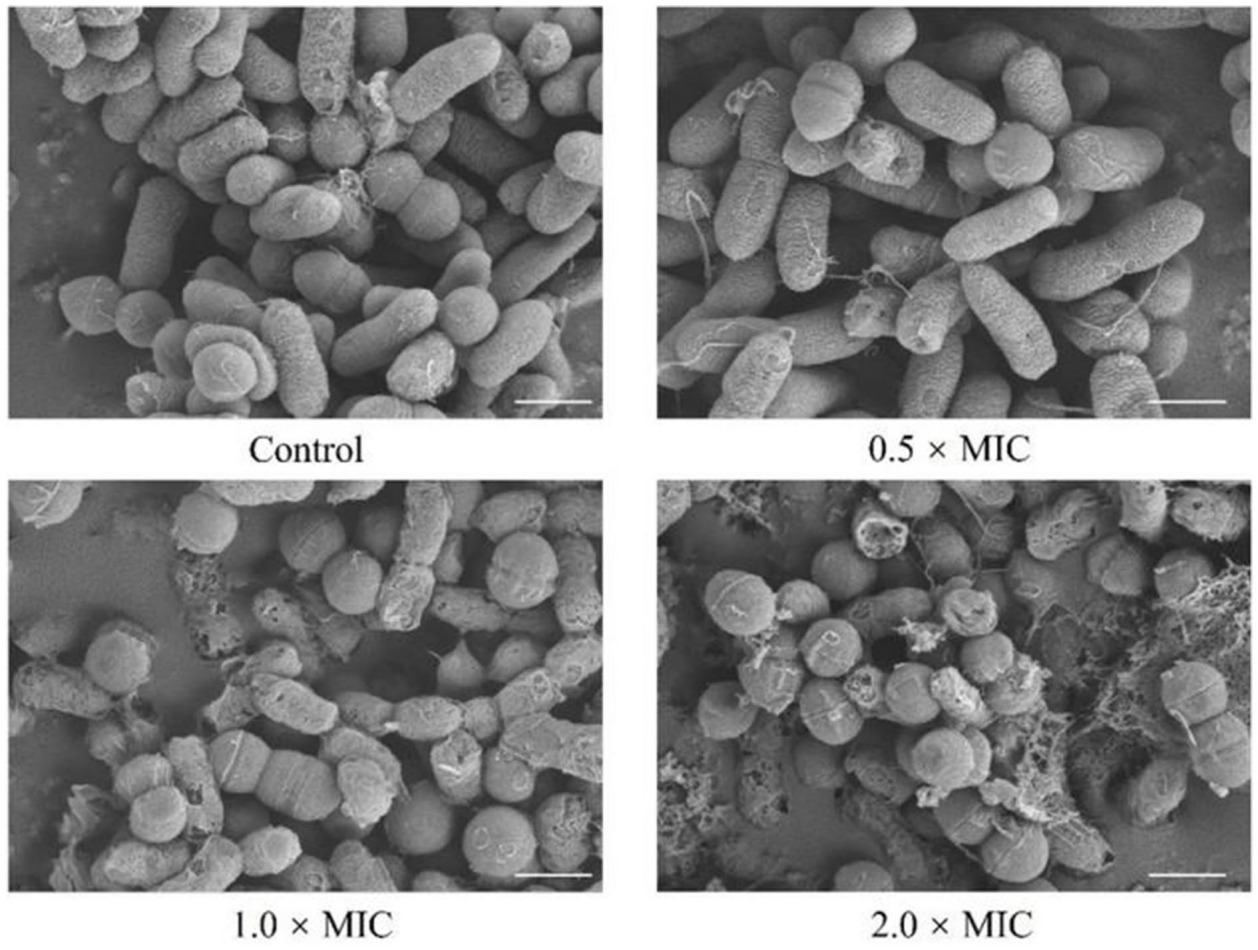
Effect of *L. plantarum* Dys01 metabolites on the morphology of *B. contaminans*. The *B. contaminans* were treated with *L. plantarum* Dys01 metabolites at final metabolite concentrations of 0.5 ×, 1 ×, and 2 × MIC. After 12 h of culture, the bacteria were subjected to field emission scanning electron microscopy analysis. The sterile deionized water was employed as a control. Scale bar, 500 nm.

To reveal the effect of *L. plantarum* Dys01 metabolites on the morphology of *B. contaminans*, field emission scanning electron microscopy was used to observe *B. contaminans* after treatment with different metabolite concentrations (Figure 7). In the untreated control, *B. contaminans* exhibited intact morphology with smooth surfaces and tight arrangement. After treatment with 0.5 × MIC of the metabolites, the bacterial surface became mild roughness and fine wrinkles began to appear. The gaps between bacteria slightly increased, and the bacteria started to exhibit a tendency toward dispersion. At 1.0 × MIC, the bacterial surfaces were severely damaged, exhibiting pore formation, significant shrinkage, deeper grooves, irregular morphology, and an increasingly loose intercellular structure. At 2.0 × MIC, significant cellular disruption and loss of morphological features were observed, with the overall morphology severely deformed and fragmented, displaying obvious cracks and signs of lysis on the bacterial surface (Figure 7). Collectively, these data indicated that the metabolites of *L. plantarum Dys01* could exert antimicrobial effects by disrupting and lysing *B. contaminans*.

## 4 Discussion

*Burkholderia contaminans*, an aerobic Gram-negative bacterium, is widely distributed in hospital environments and industrial equipment. It exhibits strong environmental adaptability and potential pathogenicity (Bennett et al., 2020; Ura et al., 2006). As an opportunistic pathogen, *B. contaminans* frequently causes severe infections such as pulmonary infections, catheter-associated infections, and urinary tract infections, especially in immunocompromised individuals (Meena et al., 2019). The remarkable biofilm-forming ability of *B. contaminans* enables stable adhesion to a variety of inert or organic surfaces and provides a structural barrier, enhancing its tolerance to antibiotics, preservatives, and other environmental stresses (Paraskevopoulos et al., 2024; Rushton et al., 2013). Therefore, novel biological antibacterial strategies using microorganisms and their metabolites have gained widespread attention, such as the use of probiotics in biopreservation. In recent years, the marine environment and the intestines of aquatic animals have been considered as important natural reservoirs of functional probiotics. These microorganisms, adapted to high-salinity, high-pressure, and nutrient-rich extreme niches, exhibit excellent environmental adaptability and complex secondary metabolic pathways, which enables them to synthesize a variety of bioactive antimicrobial metabolites. For instance, Zhang et al. (2019) isolated *L. plantarum* CLY-5 from a sea cucumber aquaculture environment, which had strong broad-spectrum antibacterial activity, particularly against *Vibrio splendidus* and *Pseudoalteromonas*. *Lactococcus lactis*, screened from fish intestines and lake water, not only exhibits a remarkable inhibitory effect against *Aeromonas hydrophila*, but also demonstrates excellent adhesion and colonization capabilities (Li et al., 2022). These studies collectively demonstrated the great potential of marine-derived lactic acid bacteria in pathogenic control and aquaculture applications. In this study, we isolated and identified *L. plantarum* Dys01 from a marine grouper intestines. This strain exhibited significant inhibitory effects against *B. contaminans* in a dose-dependent manner. Although previous studies have shown that *L. plantarum* can effectively inhibit various pathogens such as *Staphylococcus aureus*, *Escherichia coli*, and *Salmonella*, this study is the first to demonstrate its antimicrobial activity against *B. contaminans*. In this context, our findings provided new insights for the prevention and control of *B. contaminans* in food and cosmetics.

In this study, the antimicrobial activity of *L. plantarum* Dys01 against *B. contaminans* mainly resulted from the synergistic effects of its metabolites, including organic acids, proteinaceous substances and hydrogen peroxide, with organic acids having the most significant effects. As reported, the undissociated form of organic acids can penetrate bacterial membranes to acidify the cytoplasm (Zhang et al., 2011). To maintain pH homeostasis, bacteria expel H^+^, consuming ATP, and exchange potassium ions, further increasing osmotic pressure, leading to membrane rupture and leakage of intracellular contents (Zhang et al., 2011; Ji et al., 2023). In addition, the intracellular accumulation of acidic ions can interfere with or block nuclear DNA synthesis, disrupt metabolic transcription processes involved in energy production, and denature key intracellular enzymes, ultimately causing cell death (Ji et al., 2023). Some organic acids, including fruit acids, oxalic acid, and tartaric acid, block the synthesis of surface adhesion proteins, thus hindering bacterial colonization and biofilm formation (Gao et al., 2025). Bacteriocins are small proteins or peptides with broad-spectrum antibacterial activity and are commonly produced by lactic acid bacteria (Zimina et al., 2020). Generally, bacteriocins are categorized into four classes, namely Class I to Class IV. Mechanistically, bacteriocins in Class I and Class II can form channels in the target bacteria membrane, leading to the leakage of ions and small molecules, causing membrane depolarisation and cell death; some of them bind to lipid II with high affinity, blocking the transmembrane transport of peptidoglycan precursors and inhibiting cell wall synthesis (Wang et al., 2024; Zimina et al., 2020). Additionally, Class III bacteriocins can directly lyse cell wall structures, while Class IV bacteriocins embed themselves into the membrane via sugar or lipid groups, interfering with membrane enzyme activity or membrane structural stability (Darbandi et al., 2022). The wide pH stability, high thermal stability and surfactant stability of bacteriocins make them become promising natural biological preservatives (Gu et al., 2024). Hydrogen peroxide is a small and highly polar molecule, which enables it to penetrate cell membranes and integrate into phospholipids, thereby impairing membrane permeability, causing electrolyte leakage and disruption of membrane potential (Tan et al., 2025). The antibacterial activity and direct cellular damage caused by hydrogen peroxide (H₂O₂) are primarily attributed to its strong oxidative properties (Abdelshafy et al., 2024). Hydrogen peroxide generates reactive oxygen species (ROS) through the Fenton reaction, which initiates lipid peroxidation and causes severe oxidative damage to cellular components, leading to the production of toxic byproducts such as malondialdehyde (MDA) and 4-hydroxy-2-nonenal (4-HNE). These byproducts interact with intracellular proteins and nucleic acids, causing enzyme inactivation, genome damage and disruption of the integrity of cell membrane and cell wall (Abdelshafy et al., 2024). Due to the harmful effects of hydrogen peroxide and lipid peroxidation, some lactic acid bacteria secrete antioxidant enzymes such as catalase to mitigate their own sensitivity to oxidative products. Here, our study revealed that the metabolites of *L. plantarum* Dys01 inhibited growth of *B. contaminans* by suppressing biofilm formation and disrupting the integrity of cell wall and cell membrane. Given the diversity and complexity of the antimicrobials, the identification and quantification of individual antimicrobial compounds in *L. plantarum* Dys01 metabolites, as well as the further exploration of antibacterial mechanism, merit to be explored in the future. Additionally, our study was currently limited to the laboratory scale. The substantial optimization and further purification of *L. plantarum* Dys01 metabolites are required before making a comparison with commercial preservatives and antibiotics, as well as the application of these findings in industrial settings. In this context, our findings offered an important theoretical foundation for the development of novel biological antimicrobial agents in food safety and biopreservation.

## Data Availability

The raw data supporting the conclusions of this article will be made available by the authors, without undue reservation.
